# Recent Advances in Forward Brillouin Scattering: Sensor Applications

**DOI:** 10.3390/s23010318

**Published:** 2022-12-28

**Authors:** Luis A. Sánchez, Antonio Díez, José Luis Cruz, Miguel V. Andrés

**Affiliations:** Departmento de Física Aplicada y Electromagnetismo-ICMUV, Universidad de Valencia, Dr. Moliner 50, 46100 Burjassot, Spain

**Keywords:** forward Brillouin scattering, opto-mechanics, acoustic transverse resonances, fiber sensors

## Abstract

In-fiber opto-mechanics based on forward Brillouin scattering has received increasing attention because it enables sensing the surrounding of the optical fiber. Optical fiber transverse acoustic resonances are sensitive to both the inner properties of the optical fiber and the external medium. A particularly efficient pump and probe technique—assisted by a fiber grating—can be exploited for the development of point sensors of only a few centimeters in length. When measuring the acoustic resonances, this technique provides the narrowest reported linewidths and a signal-to-noise ratio better than 40 dB. The longitudinal and transverse acoustic velocities—normalized with the fiber radius—can be determined with a relative error lower than 10^−4^, exploiting the derivation of accurate asymptotic expressions for the resonant frequencies. Using this technique, the Poisson’s ratio of an optical fiber and its temperature dependence have been measured, reducing the relative error by a factor of 100 with respect to previously reported values. Using a single-point sensor, discriminative measurements of strain and temperature can be performed, achieving detection limits of ±25 με and ±0.2 °C. These results show the potential of this approach for the development of point sensors, which can be easily wavelength-multiplexed.

## 1. Introduction

The study of forward Brillouin scattering (FBS) in optical fibers, i.e., forward scattering of a guided optical wave by transverse acoustic resonances of the fiber itself, started in the 1980s [1] and is attracting increasing interest in recent years [2]. Early studies employed optical heterodyne detection to resolve the fine structure of thermally excited acoustic modes. Since then, continuous improvements in the excitation and detection approaches have impelled both fundamental studies and sensor applications. Preferable excitation schemes use either a simple optical pulse to excite a broadband of acoustic frequencies simultaneously [3], or a dual-frequency laser source for the selective excitation of acoustic resonances matching the frequency difference [4]. In both cases, electrostriction is the dominant physical effect responsible for the optical excitation of transverse acoustic resonances. Although heterodyne detection is always an option [5], detection has been carried out frequently using a Sagnac interferometer driven by an auxiliary probe signal and, alternatively, using direct high-resolution spectral measurements when the dual-frequency excitation is used [4].

Mode-locking [6] and sensing [7] are two areas in which FBS is showing a significant impact. Here, we will focus our attention on sensor applications. Liquid detection has been demonstrated by measuring cavity lifetime of radial resonances, which provides information on the reflectivity at the outer surface of the optical fiber that permits to obtain the acoustic impedance of the external medium surrounding the fiber [7]. Simultaneous measurement of relative humidity and temperature has been demonstrated in a polyimide-coated fiber, taking advantage of the dependence of the coating impedance with both humidity and temperature, and measuring the linewidth and central frequency of a given acoustic resonance [8]. Similarly, simultaneous measurement of temperature and acoustic impedance of sucrose solutions using an uncoated LEAF fiber has been reported [9].

The idea of emulating the success of distributed fiber sensing based on backward Brillouin scattering has driven some recent developments, bearing in mind that FBS would enable measuring properties of the fiber surrounding. The need for removing the coating of a standard optical fiber is certainly a severe drawback for practical applications of distributed sensors [10,11]. Typically, only some sections of the fiber are uncoated, and the reported spatial resolutions are higher than 2 m. One approach to overcome this limitation is the use of optical fibers coated with a thin layer of polyimide, since the mechanical properties of this material significantly reduce the attenuation of acoustic waves in silica fibers [12,13]. Thus, it is possible to distinguish between air, water, and ethanol outside a fiber coated with polyimide [14], with a reported resolution of 50 m. It is worth mentioning that FBS has been demonstrated to be a useful tool for the characterization of elastic properties of fiber coatings [15].

Distributed sensing based on FBS has required the development of specific measuring techniques. The first proposal used a pump and probe technique with a relatively long pump pulse and a probing system based on backwards Brillouin-stimulated scattering operated at a different wavelength than the pump [10]. Linewidth measurements were required to extract the information, achieving a 15 m spatial resolution. A technique based on Rayleigh backscattering of two optical tones coupled to a specific acoustic resonance has also been demonstrated [14], in which 1 μs pulses were used, leading to a 100 m spatial resolution. More recently, an improved pump and probe technique with a coherent stimulated probing process based on backwards Brillouin scattering has been demonstrated to significantly improve the signal-to-noise ratio (SNR) and to provide a spatial resolution of 2 m [11], which can be further reduced to 0.8 m using activation and probe signals with orthogonal polarizations [16]. An interesting alternative based on measuring the frequency shift of a short optical pulse subject to the phase chirp modulation caused by the acoustic oscillations has proven to provide a spatial resolution down to 0.8 m [17].

New protocols for pumping and distributed analysis of FBS are being reported. Using polarization-maintaining fibers, it has been proposed to pump FBS with two copropagating tones matching the orthogonal polarization axes of the fiber and to detect the acoustic resonances through the nonlinear polarization switching of a counterpropagating probe [18]. A rather different solution based on an array of chirped fiber gratings has been proposed, having 100 weak gratings along 500 m of fiber, separated by 5 m between each other. Once the acoustic resonances are excited with a harmonic-modulated long pump pulse, then a probe signal reflected in the consecutive gratings takes the information of the FBS with a spatial resolution of 5 m. Finally, the phase modulation of the probe signal is analyzed with a heterodyne coherent detection [19]. A multipoint FBS sensor has also been proposed based on frequency-division multiplexing, by using fiber sections with different diameters [20].

Regarding the weakness of the interaction which limits the achievable spatial resolution and makes the detection of FBS with a high SNR challenging, theoretical and experimental works have demonstrated several orders of magnitude enhancement at nanoscale waveguides [21]. The implementation of this solution using optical fiber technologies basically has two alternatives, either tapering a fiber down to about 1 micron [3] or using small solid-core photonic crystal fibers [4]. In both cases, sensor applications appear to be unfeasible, either because of the fragility of the tapers or because of the lack of interaction of the acoustic field with the surrounding medium due to its confinement in the core of the photonic crystal fiber.

Recently, some new concepts for sensor applications of FBS have been reported. On the one hand, a forward Brillouin fiber laser has been developed using 30 m of panda-type polarization-maintaining fiber, demonstrating its potential for sensing the surrounding of the fiber [22]. On the other hand, sensing of gamma radiation has been studied using 6–10 m-long fibers coated with thin layers of fluoroacrylate polymer [23].

Here, we will review our approach to the sensor applications of FBS, which is rather different from previously reported methods. We pay attention to the development of highly efficient point sensors, of only a few centimeters length, in which the information can be easily extracted, and the measured signal exhibits a high SNR. Instead of distributed sensing, our technique permits a straightforward and rather conventional wavelength multiplexing. First, we will present a pump and probe technique assisted by a fiber grating [24], which has the spatial resolution of the grating length and provides a high SNR. Then, we will demonstrate the potential of this technique for sensing by measuring the Poisson’s ratio of an optical fiber with an accuracy two orders of magnitude better than previously reported values [25]. Finally, we will discuss a sensor application in which simultaneous strain and temperature measurements are carried out with a single-point sensor, obtaining a resolution of 25 με and ±0.2 °C, respectively [26].

## 2. A Point Pump and Probe Technique

Sensor applications based on FBS would appear to be doomed to large spatial resolutions of the order of meters. Thus, sensing liquids with a simple drop would be beyond the achievable. The development of point sensors in which the physical mechanism for sensing the external medium is the acoustic field, but not the optical field, can give rise to a range of applications parallel and complementary to the more conventional fiber sensors based on optical mechanisms.

The technique that we present here is a development inspired by a previous method for the measurement of the nonlinear refractive index using very short lengths of optical fibers [27]. That method demonstrated the possibility of measuring the small nonlinear refractive index changes due to the Kerr effect using a pump and probe technique assisted by an acoustic grating—generated by a flexural wave—and tuned to the probe signal. Thus, the FBS technique can be described as a pump and probe technique assisted by a long-period grating (LPG), that can eventually be replaced by a fiber Bragg grating. However, the sensitivity of a fiber Bragg grating is estimated to be at least one order of magnitude lower than the sensitivity of a narrow bandwidth LPG. The pump laser excites the transverse acoustic resonances with short pulses, while the continuous wave probe signal is tuned to the side of the LPG and senses the modulation of the LPG transmittance produced by the acoustic waves via the modulation of the core refractive index.

**Figure 1:**
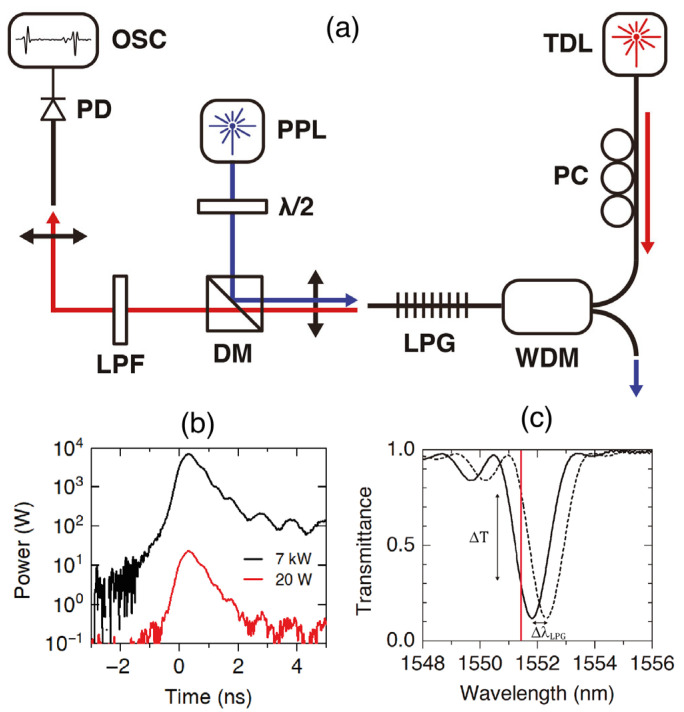
(**a**) Experimental setup. OSC: oscilloscope; PD: fast photodetector; LPF: long-pass filter; DM: dichroic mirror; WDM: wavelength division multiplexer; PPL: pulsed pump laser; λ/2: half wavelength plate; TDL: tunable continuous wave diode laser; PC: polarization controller; Blue line: pump laser path; Red line: probe laser path. (**b**) Typical pump pulses for 7 kW and 20 W peak powers. (**c**) Transmittance of the LPG and operation principle of the pump and probe technique: (i) the vertical dashed line indicates the probe laser wavelength, which is set in the linear region of the LPG transmittance, and (ii) the acoustic wave will shift the resonance wavelength of the grating (Δ*λ*_LPG_) and this will cause the transmission of the probe signal to be modulated by Δ*T*.

Figure 1a depicts our pump and probe technique. The fiber under test (FUT) has about 20 cm length and the coating was removed for two reasons, first for writing the LPG and second to have acoustic resonances with the highest possible quality factor. The FUT length could be as short as the length of the LPG (typically 10 cm). The LPG is critical for the right implementation of the technique. Since the refractive index modulation produced by the acoustic resonances can be extremely small, the LPG needs to have sharp edges to generate the highest possible transmittance modulation with small core refractive index perturbations. Thus, narrow-linewidth LPGs are required. In our case, the LPG was fabricated following a technique previously developed [28], that permits the fabrication of 1 nm bandwidth LPGs. More specifically, a high numerical aperture fiber supplied by Fibercore (SM1500-4.2/125, NA 0.29, cutoff wavelength 1387 nm) was used. The period of the grating was *Λ* = 52.3 μm, the length was *L* = 11 cm, and the notch wavelength was *λ_LPG_* = 1551 nm. The 3 dB bandwidth of the LPG was 1.3 nm and the depth of the transmittance at the resonance wavelength was −9 dB.

Figure 1b includes oscilloscope traces of the pump pulses for 7 kW and 20 W peak powers (700 ps pulse duration, 1064 nm wavelength, 19.9 kHz repetition rate). Selecting pump pulses of about 1 ns duration ensures an efficient excitation—via electrostriction [29]—of transverse acoustic resonances of hundreds of MHz. Radial resonances of order 6–8 at around 300 MHz exhibit the highest overlap with the fiber core and generate the highest modulation of the core refractive index [7].

Figure 1c includes the transmittance spectrum of the LPG and shows how a small shift of the grating will modulate the probe signal adjusted to the edge of the LPG. The experimental values of the slopes at the central point of the edges—where the slope is linear, *s* = *∂T*/*∂λ*—are *s* = −0.98 nm^−1^ for the left side of the notch and *s* = 0.90 nm^−1^ for the right side. We can estimate the relations between a small change of the core effective refractive index (*δn_co_*), a given shift of the LPG grating (*δλ_LPG_*), and a measured change of the transmittance of the probe signal (*δT*), with the expressions:

(1) $$\delta \lambda_{LPG}=\Lambda \delta n_{co}\;,\;\;\;\delta n_{co}=\frac{\delta T}{s\;\Lambda }\;.$$

In our experiments, we can measure effective refractive index changes as small as *δn_co_* = 10^−9^. However, our FBS pump and probe technique and its sensor applications do not rely on an accurate measurement of the refractive index changes, but on frequency measurements performed after a simple fast Fourier transform of the transmitted probe signal. A small refractive index detection limit will ensure a high signal-to-noise ratio (SNR).

**Figure 2:**
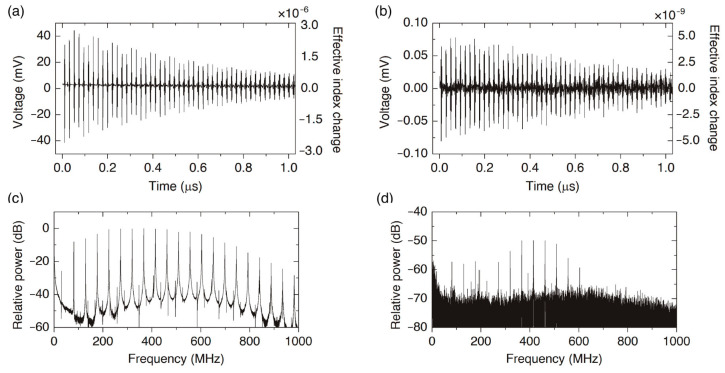
Oscilloscope traces for pump pulses of (**a**) 7 kW and (**b**) 20 W. Each one of the traces is the result of 1064 averages. (**c**,**d**) The fast Fourier transform of traces (**a**) and (**b**): SNR > 40 dB and 15 dB can be observed, respectively, for the strongest resonances.

Figure 2 shows two representative oscilloscope traces of the probe signal modulated by the acoustic waves generated with pump pulses of 7 kW and 20 W peak power, and both traces are the average of 1064 pump pulses. These plots include, on the right-hand side, the calculated effective refractive index change according to Equation (1). Figure 2b shows that effective index changes as small as 10^−9^ can be detected. The repetition frequency of the pump laser is 19 kHz—52.6 μs period—while damping of the acoustic signal, according to the oscilloscope traces, takes about 1 μs. Thus, we can ensure that the responses of two consecutive pulses are independent of each other.

A simple fast Fourier transform of the oscilloscope traces permits to obtain the spectra of the probe signals. Alternatively, a direct measurement of the spectra can be performed using a RF signal analyzer. Figure 2 includes two spectra—(c) and (d)—corresponding to the traces (a) and (b). The SNR goes from 15 to 40 dB when the pump pulse peak power increases from 20 W to 7 kW. Most works report about a 10 dB SNR. Our results can be compared with similar SNRs reported in [30], where a FUT of several meters long was used (30 m, when 1 ns pump pulses, and a Sagnac interferometer for detection), while in our experiment, only a 20 cm-long FUT was used.

**Figure 3:**
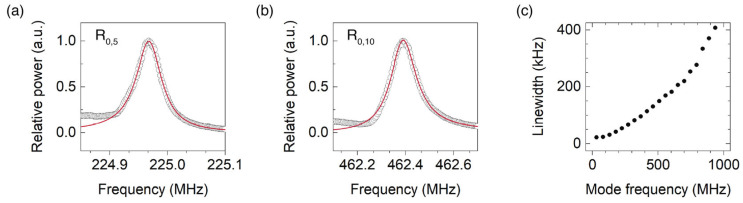
Acoustic resonances (**a**) R_0,5_ and (**b**) R_0,10_—experimental points and fitted Breit–Wigner–Fano functions—and (**c**) linewidth of R_0,m_ resonances versus the frequency.

In addition to a good SNR, the present technique provides the narrowest reported linewidths for the transverse acoustic resonances. As we will discuss later, the series of resonances observed in Figure 3 are the radial resonances, R_0,*m*_. Accurate measurement of each resonance with a RF signal analyzer permits to determine its linewidth. Figure 3 shows the spectra of resonances R_0,5_ and R_0,10_—experimental points and fitted Breit–Wigner–Fano function—and the linewidth of R_0,*m*_ modes versus their resonance frequencies. Previous studies demonstrate that the dominant contribution to the linewidth is from structural nonuniformities along the fiber in the case of uncoated fibers surrounded by air [31]. Since our technique can be implemented with only a few centimeters of fiber—20 cm in our present experiments—we are in an optimum position to demonstrate the narrowest linewidths. Some of the smallest reported linewidths are 0.1, 0.45, and 1.1 MHz for mode R_0,7_ in [10,11,14], and 80 MHz and 1.1 GHz for modes R_0,2_ and R_0,23_ in [31], while we obtained 82 kHz for mode R_0,7_, and 24 and 407 kHz for modes R_0,2_ and R_0,20_. This yields quality factors of around 3–4 × 10^3^. Sensor applications based on the measurement of liquid impedance will benefit from our pump and probe technique for both the linewidth reduction and the high SNR.

**Figure 4:**
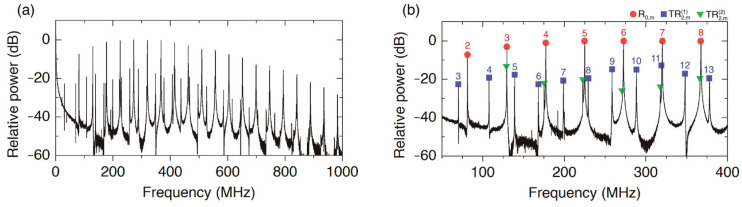
(**a**) Spectrum of transverse acoustic resonances with pump polarization adjusted for an optimum excitation of TR_2,*m*_ modes. (**b**) Detail of the strongest resonances with the identification of each resonance type: R_0,*m*_ modes are denoted by circles, while ${\mathrm{TR}}_{2,m}^{\left(1\right)}$ and ${\mathrm{TR}}_{2,m}^{\left(2\right)}$ are denoted by squares and triangles, respectively. Each resonance, R_0,*m*_ and ${\mathrm{TR}}_{2,m}^{\left(1\right)}$, is labeled with its order *m*.

One point that we have not discussed so far is the field structure of the acoustic resonances. Optical excitation of transverse acoustic resonances, either with short pulses or with dual-frequency laser sources, is limited to the excitation of radial R_0,m_ and torsional-radial TR_2,m_ resonances, with acoustic fields independent of the azimuthal angle, φ, or dependent on 2φ, respectively [1,29]. Radial resonances always exhibit the highest amplitudes, while the intensity of torsional-radial resonances depends on the pump polarization. Figure 2c illustrates the most common situation in which an efficient excitation of radial modes can be observed, while the torsional-radial modes are hardly discernible. However, readjusting the input polarization of the pump, it is possible to optimize the excitation of TR_2,m_ resonances, as it is illustrated in Figure 4. Although the intensity of torsional-radial resonances is typically about 15 dB lower than the intensity of radial resonances, they exhibit a good SNR and can be measured with high accuracy. In fact, one characteristic of the sensor applications that we propose here is to exploit both radial and torsional-radial resonances. We will show that by measuring both series of resonances, one can extract more accurate information from the sensor. For this purpose, the study presented in the next section—in which we work out high-frequency asymptotic expressions for R_0,m_ and TR_2,m_ resonances—has proven to be fundamental.

## 3. Asymptotic Expressions for High-Order Resonances

The characteristic equations for R_0,*m*_ and TR_2,*m*_ resonances are:

(2) $$R_{0,m}\;\mathrm{resonances}:\;\left(1-\alpha^{2}\right)J_{0}\left(\alpha z\right)-\alpha^{2}J_{2}\left(\alpha z\right)=0\;,$$

(3) $${\mathrm{TR}}_{2,m}\;\mathrm{resonances}:\;\left|\begin{array}{cc} \left(3-z^{2}/2\right)J_{2}\left(\alpha z\right) & \left(6-z^{2}/2\right)J_{2}\left(z\right)-3z\;J_{3}{\left(z\right)}^{}\\ \;J_{2}\left(\alpha z\right)-\alpha z\;J_{3}\left(\alpha z\right) & \left(2-z^{2}/2\right)J_{2}\left(z\right)+z\;J_{3}{\left(z\right)}_{} \end{array}\right|=0\;,$$

where *z* is the normalized frequency given by *z* = 2π*a f* /*V_S_*, and *α* = *V_S_*/*V_L_*, with *f* being the frequency, *a* the fiber radius, *V_S_* and *V_L_* the shear and longitudinal acoustic wave velocities, and *J_m_* the Bessel functions of the first kind of order, *m*.

Having in mind the idea of extracting the information from the whole spectrum of acoustic resonances, better than from one or two resonances, as it is usual in sensor applications developed so far, we have found it very useful to derive accurate asymptotic expressions for the resonance frequencies determined by Equations (2) and (3). Using Hankel’s asymptotic expansions of Bessel functions for large arguments [32], and following the procedure outlined in [25], we can derive the expressions:

(4) $$R_{0,m}\;\mathrm{resonances}:\;f_{R,m}=\frac{V_{L}}{2\pi a}\left[c_{m}-\frac{16\;\alpha^{2}-1}{8c_{m}}\right]\;,$$

(5) $${\mathrm{TR}}_{2,m}^{\left(1\right)}\;\mathrm{resonances}:\;f_{TR,m}^{\left(1\right)}=\frac{V_{S}}{2\pi a}\left[c_{m+1}-\frac{15}{8c_{m+1}}\right]\;,$$

(6) $${\mathrm{TR}}_{2,m}^{\left(2\right)}\;\mathrm{resonances}:\;f_{TR,m}^{\left(2\right)}=\frac{V_{L}}{2\pi a}\left[c_{m+1}-\frac{15}{8c_{m+1}}\right]\;,$$

where $c_{m}=m\pi -\pi /4$, *m* = 1, 2, 3, etc. These expressions retain the first two dominant terms for high-order resonances and provide high-accuracy numerical values for the frequencies of resonances, provided there is no degeneracy between ${\mathrm{TR}}_{2,m}^{\left(1\right)}$ and ${\mathrm{TR}}_{2,m}^{\left(2\right)}$ resonances.

A fundamental feature derived from Equations (4)–(6) is the splitting of the torsional-radial resonances into two series, that we denote with the superscripts (1) and (2). The asymptotic values of series (1) are determined basically by *V_S_*, while the asymptotic values of series (2) are determined by *V_L_*. In addition, series ${\mathrm{TR}}_{2,m}^{\left(2\right)}$ and $R_{0,m}$ are quasi-degenerated. Assisted by the asymptotic expressions (4)–(6), we can easily identify in an experimental spectrum to which series each resonance belongs and its order *m*, as is depicted in Figure 4b as an example.

Paying attention, in particular, to the $R_{0,m}$ and ${\mathrm{TR}}_{2,m}^{\left(1\right)}$ resonances, we have proven numerically, using the typical values for *V_L_* and *V_S_* of silica, that the above asymptotic expressions yield a relative error below 10^−3^, with respect to the exact values obtained using Equations (2) and (3), when *m* > 2 and *m* > 15, for the $R_{0,m}$ and the ${\mathrm{TR}}_{2,m}^{\left(1\right)}$, respectively. If we accept a relative error of 3 × 10^−3^, then *m* > 10 for the ${\mathrm{TR}}_{2,m}^{\left(1\right)}$ is sufficient.

Table 1 presents the experimental and theoretical frequencies for fiber SM1500, classified into the three series: $R_{0,m}$, ${\mathrm{TR}}_{2,m}^{\left(1\right)}$, and ${\mathrm{TR}}_{2,m}^{\left(2\right)}$. The theoretical values have been computed using Equations (2) and (3) and the values of *V_L_/a* = 94,463 s^−1^ and *V_S_/a* = 59,345 s^−1^ derived in Section 4.1 for T = 20 °C. This classification of the resonances in their corresponding series will be required for an optimum implementation of the sensor applications presented here. Fitting Equation (4) to the series R_0,*m*_ with *m* > 2, and fitting Equation (5) to the series ${\mathrm{TR}}_{2,m}^{\left(1\right)}$ with *m* > 15, we will obtain the normalized velocities *V_L_/a* and *V_S_/a*. The sensor information will be extracted from these values.

## 4. Sensor Applications

The first application that we will discuss is not properly a sensor application, but a measurement of a fiber parameter, the Poisson’s ratio. This application will demonstrate the potential of the present technique for the development of high-accuracy point sensors. The second application will illustrate the use of the technique for the simultaneous measurement of temperature and strain using a single-point sensor. A similar approach, but using a multi-core special fiber, harmonic modulation of the pump, and a fiber Bragg grating written in a side core, has recently been reported for detecting changes of the outer medium acoustic impedance [33]. It is worth mentioning a previous work in which FBS was used for measuring the fiber taper diameter [34] but was not developed farther as a point sensor.

### 4.1. High-Accuracy Measurement of Poisson’s Ratio

An accurate determination of Poisson’s ratio (ν) of optical fibers is an evasive issue that has been unattainable for many years. A value ranging between 0.16 and 0.17 is assumed, with a relative error of 6% [35,36]. The determination of *ν* is carried out typically by combining interferometric and polarimetric measurements. Here, we will demonstrate an accuracy improvement of about two orders of magnitude, pushing the relative error down to 10^−3^. This result proves the potential of our pump and probe approach to develop point sensors based on FBS with low detection limits.

**Figure 5:**
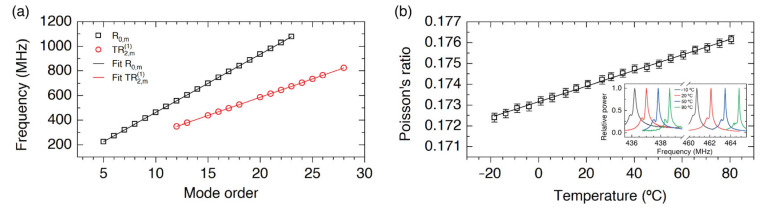
(**a**) Experimental frequencies for radial, $R_{0,m}$, and torsional-radial, ${\mathrm{TR}}_{2,m}^{\left(1\right)}$, resonances versus the order *m* at 20 °C, and the corresponding fits. (**b**) Poisson’s ratio as a function of the temperature, and a linear fit. The inset shows the RF spectra for different temperatures corresponding to resonances $R_{0,10}$ and ${\mathrm{TR}}_{2,15}^{\left(1\right)}$.

It should be mentioned that in the literature, there is a low-accuracy measurement of Poisson’s ratio using two radial-transversal resonances [37] that was pointing in the direction of exploiting FBS for the determination of *ν*. Here, by using the whole spectrum of radial, $R_{0,m}$, and torsional-radial, ${\mathrm{TR}}_{2,m}^{\left(1\right)}$, resonances, and the fitting to the asymptotic expressions derived for theses series of resonances, we achieved a rather accurate determination of *ν*. First, fitting the experimental values of the frequencies of resonance to the expressions:

(7) $$R_{0,m}\;\mathrm{resonances}:\;f_{R,m}=A\left[c_{m}-\frac{B}{8c_{m}}\right]\;,$$

(8) $${\mathrm{TR}}_{2,m}^{\left(1\right)}\;\mathrm{resonances}:\;f_{TR,m}^{\left(1\right)}=C\left[c_{m+1}-\frac{15}{8c_{m+1}}\right]\;,$$

the parameters A and C permit the determination of *V_L_*/*a* and *V_S_*/*a*, respectively. For example, at room temperature, the values that correspond to the results depicted in Figure 5a give the values *V_L_*/*a* = (94,463 ± 6) × 10^3^) s^−1^ and *V_S_*/*a* = (59,345 ± 9) × 10^3^ s^−1^. Thus, the ratio between the acoustic velocities, *α,* can be determined, and the Poisson’s ratio is obtained by means of the expression:

(9) $$\;\nu =\frac{1-2\alpha^{2}}{2\left(1-\alpha^{2}\right)}\;.$$

We find significant the fact that no length measurement is required, but only frequency measurements. Bearing in mind the reported narrow linewidths of the resonances (see Figure 3), and the good SNR, an extremely accurate determination of the frequencies can be easily performed, which leads to the determination of Poisson’s ratio with a small error: *ν* = 0.1740 ± 0.0002, at 20 °C. The good accuracy of the measurement enables the first determination of the temperature dependence of Poisson’s ratio, d*ν*/d*T*, in an optical fiber, and by repeating the measurements as a function of temperature in a temperature controlled chamber in the range from −20 to 80 °C (see Figure 5b), we obtained: d*ν*/d*T* = (3.76° ± 0.04°) × 10^−5^ °C^−1^. This value can be compared only with low-accuracy values reported for bulk silica, which range from 4 × 10^−5^ °C^−1^ to 9.6 × 10^−5^ °C^−1^ [38,39]. A more detailed comparison with the values previously reported can be found in [25]. It is worth mentioning that the relative shift of all the radial frequencies of resonance, $f_{R,m}/f_{R,m}$, with temperature is the same for large values of the order *m*, in agreement with the asymptotic expression (4), and that the same happens for the relative shift of the torsional-radial resonances, $f_{TR,m}^{\left(1\right)}/f_{TR,m}^{\left(1\right)}\;,$ according to Equation (5). The inset in Figure 5b shows the spectral shift of one radial resonance and one torsional-radial resonance, as an example.

### 4.2. Simultaneous Strain and Temperature Measurement with a Single-Point Sensor

**Figure 6:**
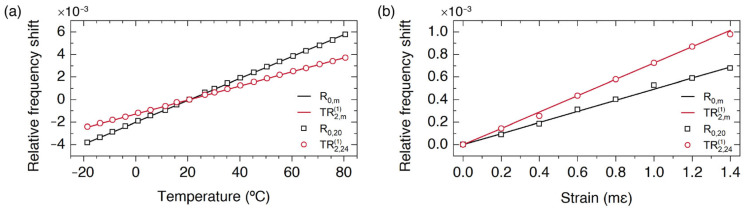
Relative frequency shift of resonances $R_{0,20}$ and ${\mathrm{TR}}_{2,24}^{\left(1\right)}$ versus temperature (**a**) and strain (**b**). Both figures include the averaged values of Δ*f/f* over all the resonances, $R_{0,m}$ and ${\mathrm{TR}}_{2,m}^{\left(1\right)}$, of each series (solid lines).

Here, we discuss the implementation of simultaneous and discriminative measurements of strain (*ε*) and temperature using a single-point sensor that exploits our FBS pump and probe technique. The proposed approach exploits the different sensitivities of radial, $R_{0,m}$, and torsional-radial, ${\mathrm{TR}}_{2,m}^{\left(1\right)}$, resonances with strain and temperature, generated by the different temperature and strain coefficients of the longitudinal and shear acoustic wave velocities (*∂V_L_/∂T* ≠ *∂V_S_/∂T* and *∂V_L_/∂ε* ≠ *∂V_S_/∂ε*). In addition, for large values of the order *m* and according to the asymptotic expressions (7) and (8), we found that the relative shift of all the resonance frequencies of radial modes versus temperature and strain, $\Delta f_{R,m}/f_{R,m}$, will be independent of the order *m*, and the same happens for the relative shift of the torsional-radial resonances, $\Delta f_{TR,m}^{\left(1\right)}/f_{TR,m}^{\left(1\right)}\;$. Thus, instead of measuring only one radial resonance and one torsional-radial resonance, it can be more robust to measure several of them, or even the whole spectrum. Figure 6 shows the relative frequency shift of two specific resonances and the averaged value obtained using the whole spectrum, showing that there is a perfect agreement. From these measurements, we can calibrate the sensor and obtain the temperature and strain coefficients for $\Delta f_{R}/f_{R}$ and $\Delta f_{TR}/f_{TR}$ defined by the elements $c_{R,TR}^{\varepsilon ,T}$ of the following 2 × 2 matrix:

(10) $$\left[\begin{array}{c} \Delta f_{R}^{}/f_{R}\\ \Delta f_{TR}^{}/f_{TR} \end{array}\right]=\left[\begin{array}{cc} c_{R}^{\varepsilon } & c_{R}^{T}\\ c_{TR}^{\varepsilon } & c_{TR}^{T} \end{array}\right]\left[\begin{array}{c} \Delta \varepsilon \\ \Delta T \end{array}\right]\;.$$

The experimental values that we obtained for this calibration are summarized in Table 2. This table also includes the elements of the inverse matrix, $s_{R,TR}^{\varepsilon ,T}$, which permits to obtain the increments of strain and temperature for a given measurement of the relative shifts. For example, when simultaneous strain and temperature increments were applied and the relative frequency shifts were measured for resonances $R_{0,10}$, $R_{0,20}$, ${\mathrm{TR}}_{2,15}^{\left(1\right)}$, and ${\mathrm{TR}}_{2,24}^{\left(1\right)}$, giving an average $\Delta f_{R,m}/f_{R,m}=4.445\times 10^{-3}$, and $\Delta f_{TR,m}^{\left(1\right)}/f_{TR,m}^{\left(1\right)}=3.227\times 10^{-3}\;$, using the coefficients $s_{R,TR}^{\varepsilon ,T},$ we obtained Δ*ε* = 898 × 10^−6^ and Δ*T* = 41.2 °C, while the actual strain and temperature increments applied to the sensor were 900 ± 10 με and 41.4 ± 0.1 °C. As it can be observed, a good agreement was attained.

Following the procedure described in [40], the errors of the strain and temperature increments, *e*_Δ*T*_ and *e*_Δ*ε*_, should be determined following the expressions:

(11) $$e_{\Delta T}=\frac{\sqrt{{\left(c_{TR}^{\varepsilon }\cdot e_{R}\right)}^{2}+{\left(c_{R}^{\varepsilon }\cdot e_{TR}\right)}^{2}}}{\left|c_{TR}^{T}\cdot c_{R}^{\varepsilon }-c_{TR}^{\varepsilon }\cdot c_{R}^{T}\right|}\;\;\;\;\;\;\;\;\;e_{\Delta \varepsilon }=\frac{\sqrt{{\left(c_{TR}^{T}\cdot e_{R}\right)}^{2}+{\left(c_{R}^{T}\cdot e_{TR}\right)}^{2}}}{\left|c_{TR}^{T}\cdot c_{R}^{\varepsilon }-c_{TR}^{\varepsilon }\cdot c_{R}^{T}\right|}\;,$$

where $e_{R}$ and $e_{TR}$ are the errors of the measured relative frequency shifts of radial and torsional-radial resonances. In our experiments, since the typical value for $e_{R}$ and $e_{TR}$ is 9 × 10^−6^, we can determine the strain and temperature accuracies using Expression (11): ±25 με and ±0.2 °C. A detailed comparison with the performance of different methods presented in the literature can be found in [26].

## 5. Conclusions

FBS point sensors based on the pump and probe technique presented here have a great potential since the measured resonances exhibit high SNR and the narrowest reported linewidths. The required signal processing is a simple fast Fourier transform. A set of wavelength-multiplexed point sensors can be an interesting alternative to distributed sensing based on FBS, which requires rather more complex experimental arrangements and signal processing than the point sensors. Since fiber coating is still a limitation for the development of an FBS-distributed sensor, wavelength-multiplexed point sensors requiring only short sections of uncoated fiber provide a practical alternative.

The asymptotic expressions presented here permit the analysis of the whole spectrum of acoustic resonances to extract accurate information on the longitudinal and shear acoustic velocities, using a simple and standard least-squares fit. Thus, sensor and fiber characterization applications will benefit from this new possibility to improve their performance. Furthermore, sensor applications that are based on the changes of the acoustic impedance of the outer fiber medium and rely on measuring the linewidth of the acoustic resonances will benefit from the small linewidths reported here, enabling lower detection limits. These improvements shall drive the development of environmental sensors and new applications in biosensing.

Future development should pay particular attention to long-range sensing and harsh conditions, which have not been tested yet.

## Figures and Tables

**Table 1:** Experimental and theoretical values of the frequencies of resonance classified into the series $R_{0,m}$, ${\mathrm{TR}}_{2,m}^{\left(1\right)}$, and ${\mathrm{TR}}_{2,m}^{\left(2\right)}$, according to their asymptotic behavior.

*m*	R_0,*m*_ (MHz)		TR_2,*m*_^(1)^ (MHz)		TR_2,*m*_^(2)^ (MHz)	
	Experiment	Theory	Experiment	Theory	Experiment	Theory
1	30.17	29.98	39.16	39.02	22.20	22.14
2	81.23	80.85	80.26	80.80	70.10	69.87
3	129.44	128.84	107.73	107.55	125.68	125.03
4	177.24	176.43	139.02	138.82	175.22	174.49
5	224.83	223.89	168.17	167.82	222.48	221.53
6	272.40	271.29	198.85	198.59	271.04	269.68
7	319.89	318.65	229.20	228.86	317.56	316.41
8	367.32	365.98	258.39	258.25	366.30	364.72
9	414.76	413.31	288.38	288.25	414.43	412.34
10	462.13	460.62	319.32	318.1	461.74	459.57
11	509.51	507.93	347.94	347.76	509.10	507.06
12	556.82	555.23	377.77	377.61	556.36	556.19
13	604.08	602.53	407.07	407.16	603.57	601.77
14	651.42	649.82	437.01	437.04	650.86	649.20
15	698.68	697.12	466.72	466.86	698.06	696.44
16	745.99	744.41	496.51	496.47	745.27	743.81
17	793.28	791.70	526.19	526.24	792.53	793.69
18	840.51	838.98	554.80	554.18	839.63	838.44
19	887.77	886.27	585.41	585.66	886.82	885.81
20	935.03	933.56	615.20	615.40		
21	982.25	980.84	644.86	645.02		
22	1029.54	1028.12	674.69	674.80		
23	1076.72	1075.41	704.84	704.53		
24	1123.98	1122.69	733.81	734.19		
25	1171.34	1169.97	763.64	763.92		
26			--	791.05		
27			823.09	823.30		
28			852.89	853.02		
29			881.88	882.66		

**Table 2:** Calibration of the sensor: $c_{R,\mathrm{TR}}^{\varepsilon ,T}$ and $s_{R,TR}^{\varepsilon ,T}$ coefficients.

$c_{R}^{\varepsilon }$ (με^−1^)	$c_{R}^{T}$ (°C^−1^)	$c_{TR}^{\varepsilon }$ (με^−1^)	$c_{TR}^{T}$ (°C^−1^)
4.82 × 10^−7^	9.73 × 10^−5^	7.25 × 10^−7^	6.24 × 10^−5^
$s_{R}^{\varepsilon }$ **(με)**	$s_{R}^{T}$ **(°C)**	$s_{TR}^{\varepsilon }$ **(με)**	$s_{TR}^{T}$ **(°C)**
−0.154 × 10^7^	0.179 × 10^5^	0.240 × 10^7^	−0.119 × 10^5^

## Data Availability

Data will be made available upon reasonable request.
